# The Complex Hemodynamic Interplay between Mitral Arcade, Midventricular Obstruction, and Prosthetic Aortic Valve

**DOI:** 10.1016/j.case.2024.02.003

**Published:** 2024-03-25

**Authors:** Giovanni Taverna, Antonino Micari, Olimpia Trio, Tommaso D'Angelo, Gianluca Di Bella, Francesco F. Faletra, Giuseppe Andò

**Affiliations:** a Department of Clinical and Experimental Medicine, University of Messina and Azienda Ospedaliera Universitaria Policlinico “Gaetano Martino”, Messina, Italy; b Department of Biomedical and Dental Sciences and Morphological and Functional Imaging, University of Messina and Azienda Ospedaliera Universitaria Policlinico “Gaetano Martino”, Messina, Italy; c Istituto Mediterraneo per i Trapianti e Terapie ad Alta Specializzazione—University of Pittsburgh Medical Center, Palermo, Italy

**Keywords:** Mitral arcade, Transthoracic echocardiography, Transesophageal echocardiography, Cardiac computed tomography, Multimodal imaging

## Abstract

• MA is a malformation of valve tensor apparatus leading to early heart failure.
• MA can be associated with subvalvar aortic stenosis, bicuspid AV, or aortic coarctation.
• Multimodal imaging is invaluable in assessing mitral arcade.

## Graphical abstract

**Figure:**
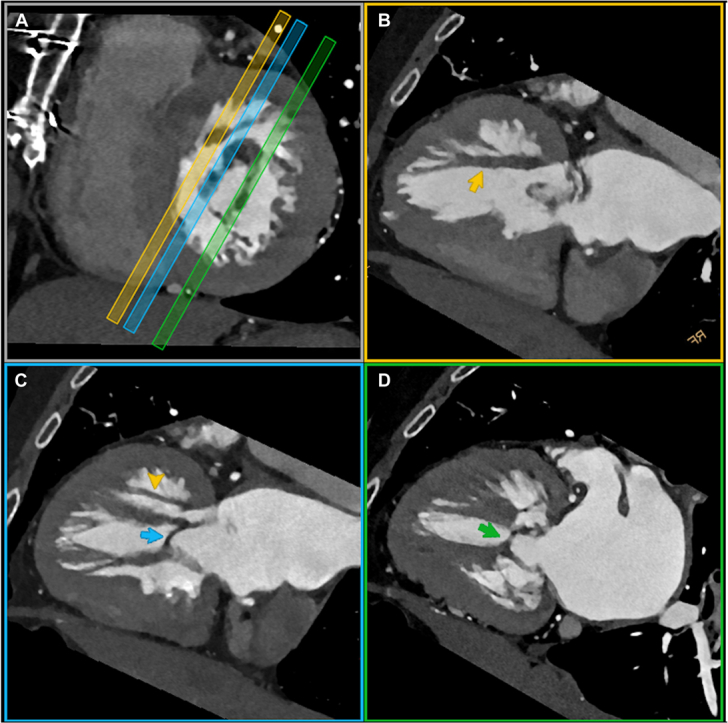

## Introduction

Mitral arcade (MA) is an uncommon congenital malformation of the tensor apparatus of the mitral valve (MV) first described by Layman and Edwards in 1967.^1^ It consists of enlarged and elongated papillary muscles connected to each other and to the free edge of the anterior mitral leaflet by a bridge of fibrous tissue, which resembles an arcade.^2^

We report here the case of a patient with 2 bridges of fibrous tissue between the tips of the 2 papillary muscles consistent with MA. Such valvular anomaly can be associated with other obstructive left heart lesions including bicuspid aortic valve (AV), subaortic stenosis, or coarctation of the aorta.

## Case Presentation

A 55-year-old woman was referred because of worsening New York Heart Association class III dyspnea and poor exercise tolerance. Clinical examination revealed a multifocal Levine grade 3/6 systolic murmur and a crisp metallic click after the carotid pulse. A 12-lead electrocardiogram demonstrated normal sinus rhythm at 60 bpm, left ventricular (LV) hypertrophy, and atrial enlargement. The patient denied any history of atrial arrhythmias. At the age of 19 the patient had been diagnosed with bicuspid AV and fibromuscular subvalvular aortic stenosis and at the age of 39 had undergone surgical AV replacement with a 19 mm mechanical prosthesis and resection of the fibromuscular subaortic membrane.

**Figure 1:**
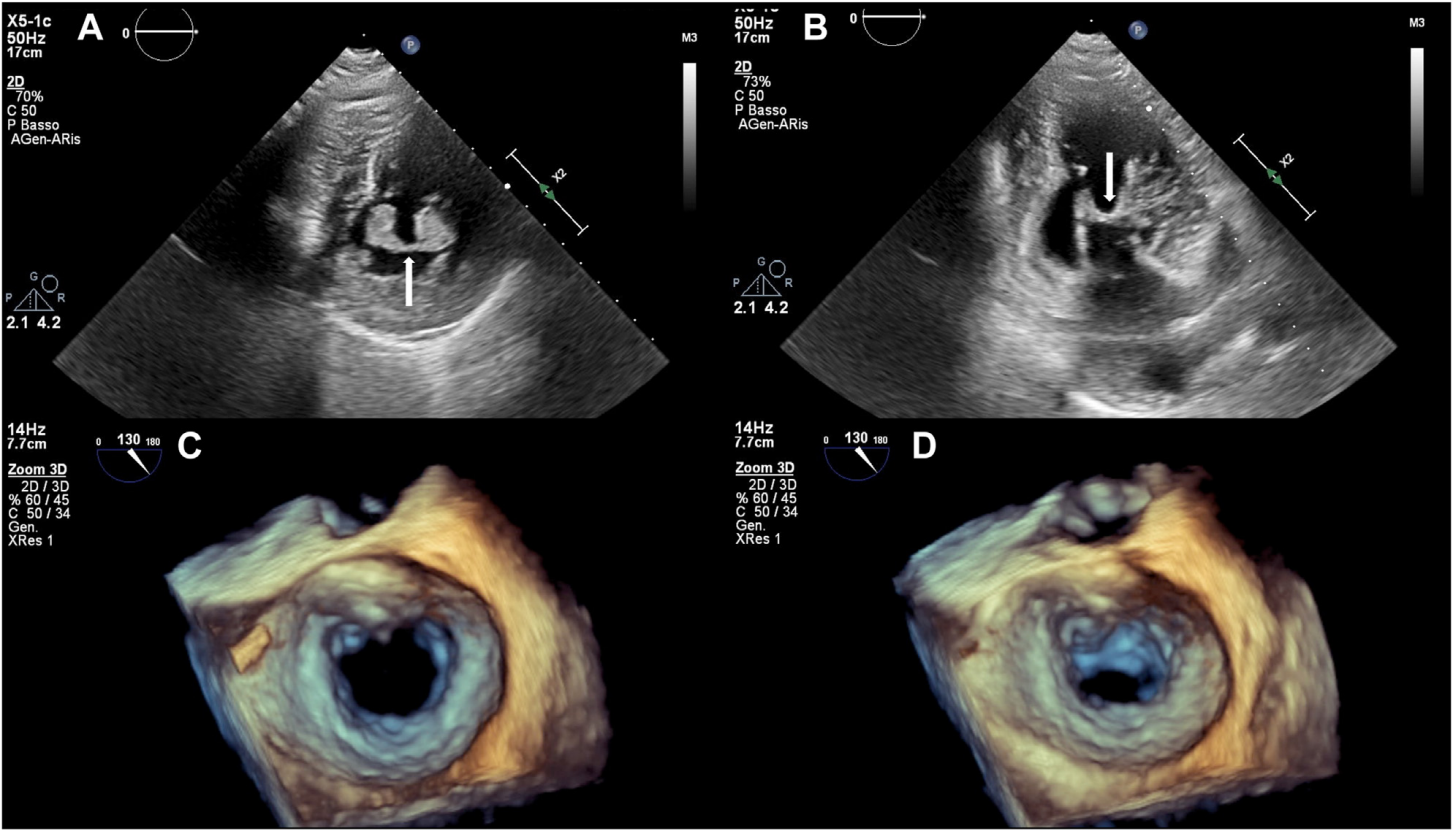
Two-dimensional TTE, parasternal short-axis **(A)** and apical 2-chamber **(B)** diastolic views demonstrate the anomalous fibrous bands (*arrows*) connecting the 2 hypertrophic papillary muscles. Midesophageal three-dimensional TEE, volume-rendered en face diastolic views of the MV from the left atrial **(C)** and LV **(D)** perspective demonstrate the normal opening of the leaflets and the narrowed subvalvular orifice.

**Figure 2:**
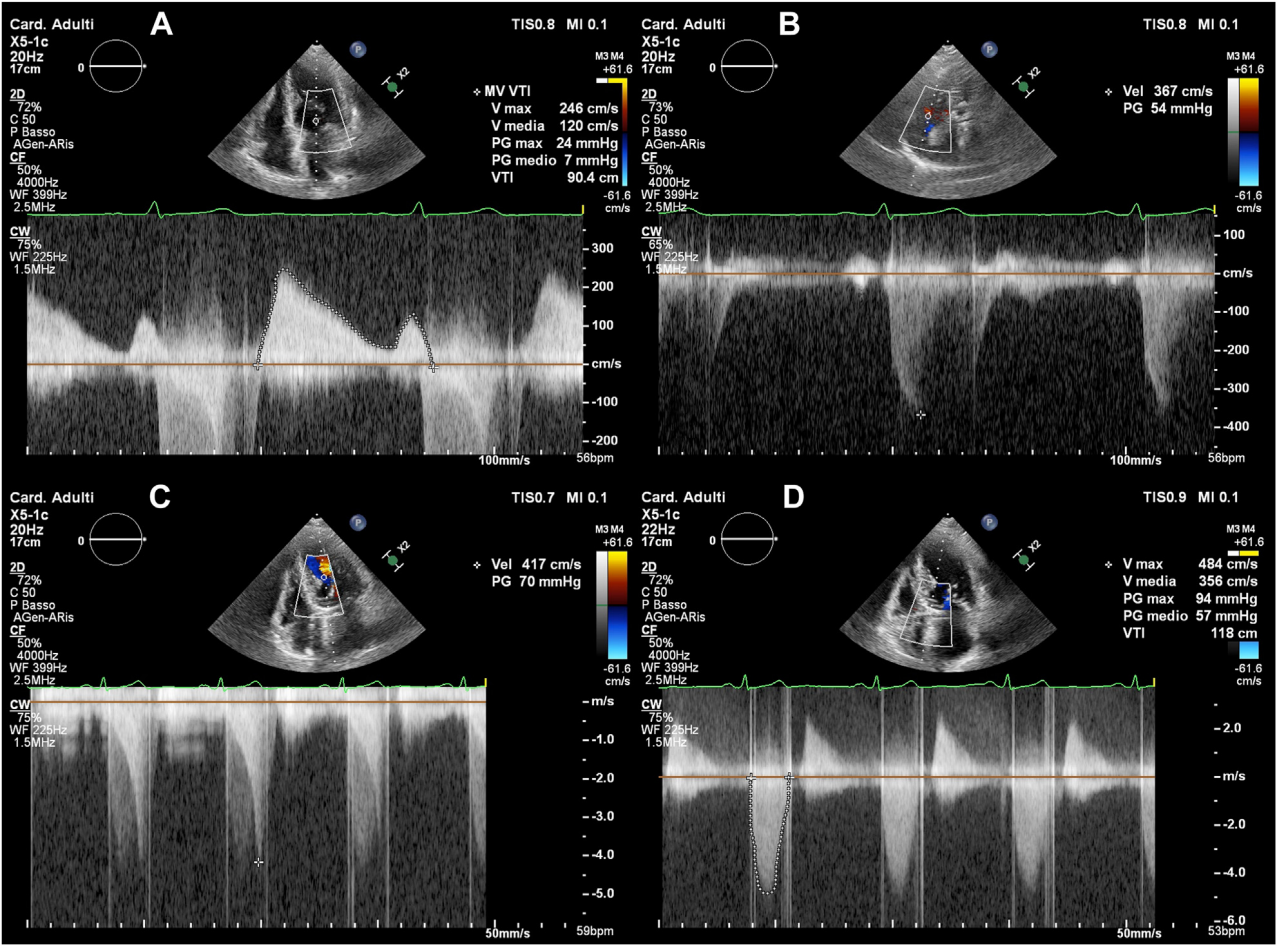
Two-dimensional TTE-guided continuous-wave Doppler spectral profiles from the apical 4-chamber view demonstrate **(A)** the diastolic mean gradient (7 mm Hg) across the MV; **(B)** the maximal tricuspid regurgitation velocity (367 cm/sec) and estimated gradient (54 mm Hg); **(C)** the “dagger-shaped” midventricular functional systolic resting gradient (70 mm Hg) across the LV outflow tract; and **(D)** the peak (94 mm Hg) and mean (57 mm Hg) fixed systolic gradients across the aortic prosthesis.

The patient underwent a comprehensive workup including transradial^3^ coronary angiography, transthoracic echocardiography (TTE), and transesophageal echocardiography (TEE). Short and thick chordae with almost direct attachment of elongated and hypertrophic papillary muscles to mitral leaflets were evident; mitral leaflets were mildly thickened, and a normal orifice area was observed at the valvular level (Figure 1, Video 1, Video 2, Video 3, Video 4, Video 5). No commissural fusions or calcifications were found, but 2 fibrous bands extended between papillary muscles tips, with an arcade-like configuration in the short-axis view. Moderate stenosis (the mean gradient was 7 mm Hg with a heart rate of 56 bpm) and moderate mitral regurgitation (MR; vena contracta diameter and area were 0.6 cm and 0.31 cm^2^, respectively) were observed, but the site of the flow abnormalities was the subvalvular apparatus (Figure 2A, Video 4). The left atrium was markedly enlarged (97 mL, 67 mL/m^2^), the right ventricle was normal, and systolic pulmonary artery pressure was estimated to be 64 mm Hg (Figure 2B). Normal LV ejection fraction and concentric hypertrophy (septum 13 mm, posterior wall 12 mm) were evident, with no residual or recurrent subvalvar membrane. Severe left midventricular obstruction was caused by a muscular bundle connecting the apex and the basal anterior septum; the typical “dagger-shaped” midventricular gradient at rest was 70 mm Hg with late peaking curve (Figure 2C). Importantly, we also observed elevated aortic prosthesis gradients, with peak and mean values of 94 and 57 mm Hg, respectively, exhibiting a distinct fixed-gradient shape (Figure 2D). Coarctation of the aorta was ruled out (Video 6). Following the proposed algorithm for prosthetic heart valve assessment,^4^ our findings indicated subvalvular acceleration as the sole mechanism for the heightened transprosthetic gradient. Confirmation of normal prosthesis function through fluoroscopy supported this conclusion.

**Figure 3:**
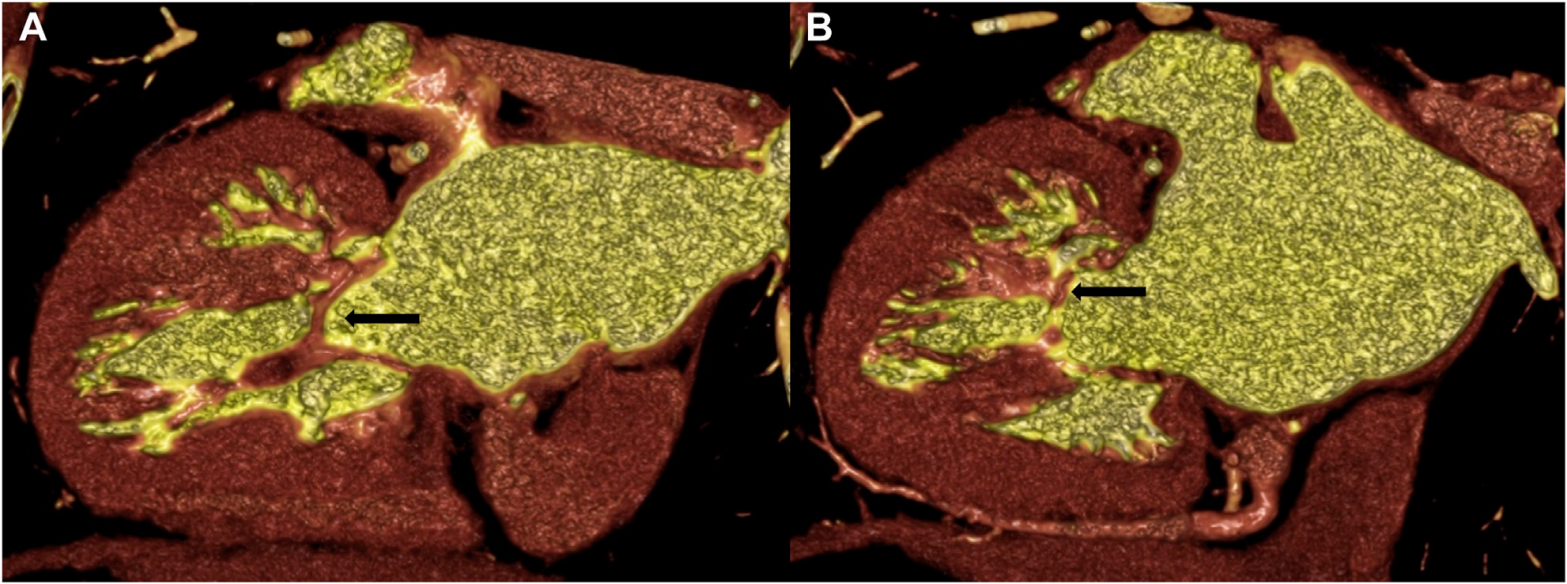
Volume-rendered, three-dimensional cardiac computed tomography (CCT) reconstructions performed along 2 vertical long-axis diastolic views demonstrate the fibromuscular bands (*arrows*) on a medial **(A)** and lateral **(B)** location.

**Figure 4:**
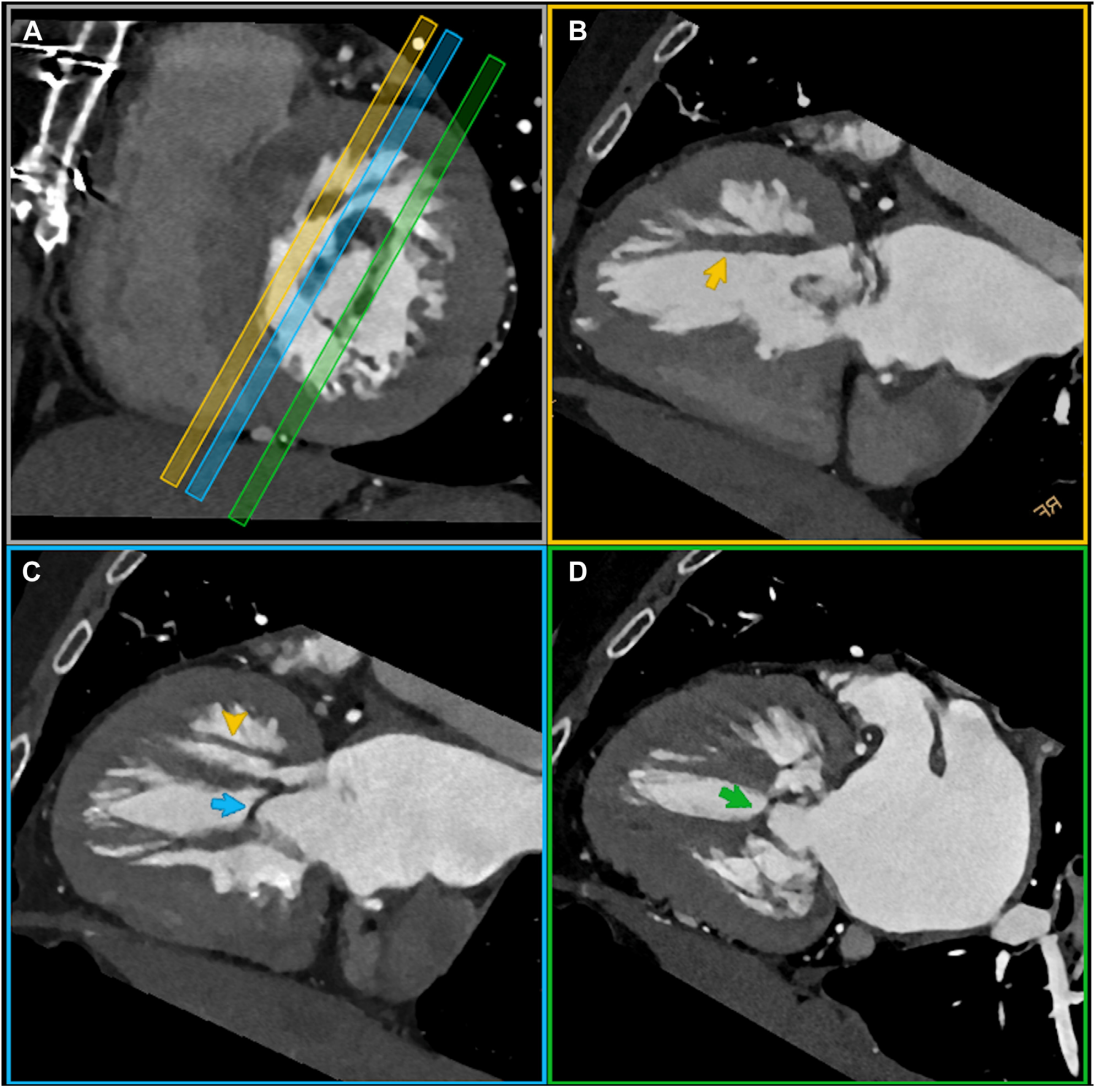
Multiplanar, basal short axis–guided **(A)**, vertical long axis **(B-D)** cardiac computed tomography reconstructions through the pathologic mitral apparatus demonstrate the subvalvular orifice (1.4 cm^2^) defined anteriorly by the anterolateral papillary muscle, posteriorly by the tip of posteromedial papillary muscle, medially (*blue slice*), and laterally (*green slice*) by 2 fibrous bands (*blue and green arrows*). An accessory muscular bundle, parallel to the anterolateral papillary muscle and running from the apex to the anterior basal septum close to the mitral annulus, is best demonstrated in a more medially aligned reconstruction (*yellow slice*) and further highlighted in panels B (*yellow arrow*) and C (*yellow arrowhead*).

Further investigation with electrocardiogram-gated cardiac computed tomography was performed to assess valvular pathoanatomy. Normal motion of aortic mechanical occluders and no evidence of pannus or thrombus were confirmed as well as the findings on the mitral subvalvular apparatus (Figures 3 and 4, Video 7, Video 8); the orifice area of 1.4 cm^2^ was consistent with a moderate stenosis.

Considering symptoms and hemodynamic consequences of both MV malformation and residual midventricular obstruction despite previous resection of the subvalvular membrane, the indication for a re-do surgery was given. The patient refused any further surgical procedure, and medical management was aimed at decongestion, adding furosemide (25 mg), and initiation of small doses of neurohumoral blockers (bisoprolol 1.25 mg and valsartan 40 mg). At 1-month telephonic follow-up, the patient reported an improvement in exercise tolerance.

## Discussion

Mitral arcade is among the least common congenital MV abnormalities. It was first described in 1967 by Layman and Edwards after postmortem evaluation of 3 newborns with MR who died suddenly.^1^ Few cases have been reported thereafter, and most of them were in children; MA is much rarer in adults.^5^, ^6^, ^7^, ^8^, ^9^, ^10^, ^11^, ^12^ Indeed, the condition is often evident early after birth, and clinical presentation consists of overt heart failure^1^ with early mortality and extremely rare survival into adulthood; milder forms can be asymptomatic and incidentally diagnosed later.

The main features of MA are adequately sized MV orifice, elongated papillary muscles, and short thickened chordae; in the most serious cases the interchordal spaces are completely obliterated so as to cause a direct connection between the papillary muscles and between the latter and the free edge of the anterior leaflet of the MV, hence forming a bridge of fibromuscular tissue, which resembles an arcade below the MV. In the classical MA^1^ the posterior mitral leaflet is spared and has preserved mobility, although a direct anomalous connection between the papillary muscles and the posterior mitral leaflet has been described.^10^ Narrowing of the interchordal spaces causes a restricted leaflet motion, mainly of the anterior one. Leaflet coaptation is therefore impaired, and this generates MR. At the same time, the reduced width or the obliteration of the interchordal spaces and the bridge connecting papillary muscles obstruct diastolic blood flow toward the apex, causing subvalvular stenosis. Mitral arcade may indeed cause variable degrees of valvular stenosis, regurgitation, or both.

Several peculiarities of this clinical case are worth mentioning. This 55-year-old woman had already undergone open-heart surgery consisting of bicuspid AV replacement and subvalvular aortic membrane resection, while concomitant MV abnormality had possibly been unrecognized, or less significant. Nonetheless, a residual disease, now consisting of MR and 3 serial obstructions, had been demonstrated. These sequential left-sided inflow and outflow obstructive lesions allow this case to be considered a partial Shone's complex.^13^ The first obstruction is at ventricular inflow due to congenital mitral stenosis caused by MA. The MA pathoanatomy is atypical,^1^ consisting of a double bridge connecting both the anterior and posterior leaflets to the papillary muscles, resulting in a stenotic mitral subvalvular orifice. The second site of obstruction is within the ventricle, involving an accessory muscular bundle. The third obstruction is observed across the AV. During this evaluation, the gradient is described as functional and the AV prosthetic occluders demonstrated normal motion. The elevated transprosthetic gradient was attributed to the accelerated flow demonstrated in the LV outflow tract.^4^

## Conclusion

The small number of reported cases in adults highlights that MA is a rare and possibly underdiagnosed malformation. Careful echocardiographic evaluation, seeking the features described by Layman and Edwards^1^ and by Hakim *et al.*,^10^ should be performed. Differential diagnosis is crucial in patients exhibiting restrictive leaflet movement not attributable to rheumatic etiology or when the obstruction site is in the subvalvular apparatus. While the high spatial resolution of cardiac computed tomography provides a detailed view of the pathoanatomical features of chordae and papillary muscles, it does not allow for the assessment of valvular hemodynamics. Therefore, a comprehensive multimodal cardiac imaging approach is essential for identifying MA from both anatomical and pathophysiological perspectives.

## Ethics Statement

The authors declare that the work described has been carried out in accordance with The Code of Ethics of the World Medical Association (Declaration of Helsinki) for experiments involving humans.

## Consent Statement

Complete written informed consent was obtained from the patient (or appropriate parent, guardian, or power of attorney) for the publication of this study and accompanying images.

## Funding Statement

This work was institutionally supported by the University of Messina through the agreement between Elsevier and the CRUI and through the APC initiative.

## Disclosure Statement

The authors report no conflict of interest.

## Supplementary Data

Video 1 Two-dimensional TTE, parasternal short-axis view, demonstrates the anomalous fibrous bands connecting the 2 hypertrophic papillary muscles.

Video 2 Two-dimensional TTE, apical 2-chamber view, demonstrates the anomalous fibrous bands connecting the 2 hypertrophic papillary muscles.

Video 3 Midesophageal, two-dimensional TEE, oblique long-axis views (128°) without and without color-flow Doppler, demonstrates the MV anatomy and moderate MR.

Video 4 Midesophageal three-dimensional TEE, volume-rendered en face view of the MV from the left atrial perspective, demonstrates the normal opening of the mitral leaflets (and the narrowed subvalvular orifice in the distance).

Video 5 Midesophageal three-dimensional TEE, volume-rendered en face view of the MV from the LV perspective, demonstrates the narrowed subvalvular orifice (and the normal opening of the mitral leaflets in the distance).

Video 6 Two-dimensional TTE, suprasternal long-axis view with color-flow Doppler of the aortic isthmus, demonstrates normal flow velocity in the descending aorta without evidence for a coarctation.

Video 7 Dynamic cardiac computed tomography, whole-cycle reconstruction, long-axis 3-chamber view, demonstrates the normal opening of the prosthetic AV and the midventricular systolic obstruction.

Video 8 Three-dimensional cardiac computed tomography, serial volume-rendered vertical long-axis reconstructions displayed medially to laterally, comprehensively demonstrates the anomalous subvalvular mitral apparatus.
